# Supplementary material for: Pyruvate Dehydrogenase Kinase 1 inhibition mediated oxidative phosphorylation enhancement in cartilage promotes osteoarthritis progression
Source: BMC Musculoskelet Disord. 2023 Jul 20;24:597. doi: 10.1186/s12891-023-06585-6 (PMC10357736; doi:10.1186/s12891-023-06585-6)
Supplement: Supplementary file 1 — Additional file 1. [file 12891_2023_6585_MOESM1_ESM.zip › MF.Gsea.1653627316242/GOMF_TAU_PROTEIN_KINASE_ACTIVITY.html]

Details for gene set GOMF\_TAU\_PROTEIN\_KINASE\_ACTIVITY[GSEA]

|  || Dataset | 55457\_collapsed\_to\_symbols.cls.cls #OA\_versus\_normal.cls.cls #OA\_versus\_normal\_repos |
| Phenotype | cls.cls#OA\_versus\_normal\_repos |
| Upregulated in class | normal |
| GeneSet | GOMF\_TAU\_PROTEIN\_KINASE\_ACTIVITY |
| Enrichment Score (ES) | -0.66869986 |
| Normalized Enrichment Score (NES) | -2.0402725 |
| Nominal p-value | 0.0 |
| FDR q-value | 0.009234663 |
| FWER p-Value | 0.035 |
Table: GSEA Results Summary

  

Fig 1: Enrichment plot: GOMF\_TAU\_PROTEIN\_KINASE\_ACTIVITY      
 Profile of the Running ES Score & Positions of GeneSet Members on the Rank Ordered List

  

| SYMBOL | TITLE | RANK IN GENE LIST | RANK METRIC SCORE | RUNNING ES | CORE ENRICHMENT || 1 | CDK5 | cyclin dependent kinase 5 [Source:HGNC Symbol;Acc:HGNC:1774] | 3400 | 0.211 | -0.2351 | No |
| 2 | CSNK1D | casein kinase 1 delta [Source:HGNC Symbol;Acc:HGNC:2452] | 4529 | 0.111 | -0.3075 | No |
| 3 | SIK3 | SIK family kinase 3 [Source:HGNC Symbol;Acc:HGNC:29165] | 5476 | 0.035 | -0.3762 | No |
| 4 | BRSK2 | BR serine/threonine kinase 2 [Source:HGNC Symbol;Acc:HGNC:11405] | 6238 | -0.023 | -0.4321 | No |
| 5 | PHKG1 | phosphorylase kinase catalytic subunit gamma 1 [Source:HGNC Symbol;Acc:HGNC:8930] | 6451 | -0.039 | -0.4432 | No |
| 6 | DYRK1A | dual specificity tyrosine phosphorylation regulated kinase 1A [Source:HGNC Symbol;Acc:HGNC:3091] | 7667 | -0.132 | -0.5195 | No |
| 7 | MARK4 | microtubule affinity regulating kinase 4 [Source:HGNC Symbol;Acc:HGNC:13538] | 9590 | -0.282 | -0.6301 | Yes |
| 8 | MARK2 | microtubule affinity regulating kinase 2 [Source:HGNC Symbol;Acc:HGNC:3332] | 9662 | -0.288 | -0.5962 | Yes |
| 9 | FYN | "FYN proto-oncogene, Src family tyrosine kinase [Source:HGNC Symbol;Acc:HGNC:4037]" | 10092 | -0.330 | -0.5843 | Yes |
| 10 | ROCK2 | Rho associated coiled-coil containing protein kinase 2 [Source:HGNC Symbol;Acc:HGNC:10252] | 10648 | -0.388 | -0.5743 | Yes |
| 11 | TAOK2 | TAO kinase 2 [Source:HGNC Symbol;Acc:HGNC:16835] | 10718 | -0.395 | -0.5256 | Yes |
| 12 | PHKG2 | phosphorylase kinase catalytic subunit gamma 2 [Source:HGNC Symbol;Acc:HGNC:8931] | 10830 | -0.408 | -0.4783 | Yes |
| 13 | MARK1 | microtubule affinity regulating kinase 1 [Source:HGNC Symbol;Acc:HGNC:6896] | 11031 | -0.434 | -0.4345 | Yes |
| 14 | MARK3 | microtubule affinity regulating kinase 3 [Source:HGNC Symbol;Acc:HGNC:6897] | 11063 | -0.440 | -0.3767 | Yes |
| 15 | GSK3B | glycogen synthase kinase 3 beta [Source:HGNC Symbol;Acc:HGNC:4617] | 11157 | -0.451 | -0.3222 | Yes |
| 16 | PRKAA1 | protein kinase AMP-activated catalytic subunit alpha 1 [Source:HGNC Symbol;Acc:HGNC:9376] | 11499 | -0.501 | -0.2802 | Yes |
| 17 | ROCK1 | Rho associated coiled-coil containing protein kinase 1 [Source:HGNC Symbol;Acc:HGNC:10251] | 12046 | -0.605 | -0.2398 | Yes |
| 18 | TTBK2 | tau tubulin kinase 2 [Source:HGNC Symbol;Acc:HGNC:19141] | 12083 | -0.615 | -0.1585 | Yes |
| 19 | GSK3A | glycogen synthase kinase 3 alpha [Source:HGNC Symbol;Acc:HGNC:4616] | 12384 | -0.703 | -0.0856 | Yes |
| 20 | TAOK1 | TAO kinase 1 [Source:HGNC Symbol;Acc:HGNC:29259] | 12753 | -0.918 | 0.0114 | Yes |
Table: GSEA details [plain text format]

  

Fig 2: GOMF\_TAU\_PROTEIN\_KINASE\_ACTIVITY      
 Blue-Pink O' Gram in the Space of the Analyzed GeneSet

  

Fig 3: GOMF\_TAU\_PROTEIN\_KINASE\_ACTIVITY: Random ES distribution      
 Gene set null distribution of ES for **GOMF\_TAU\_PROTEIN\_KINASE\_ACTIVITY**
